# Brain nuclei and neural circuits in neuropathic pain and brain modulation mechanisms of acupuncture: a review on animal-based experimental research

**DOI:** 10.3389/fnins.2023.1243231

**Published:** 2023-08-30

**Authors:** Na Su, Pingping Cai, Zhiqiang Dou, Xiaoxue Yin, Hongmin Xu, Jing He, Zhaofeng Li, Changzhong Li

**Affiliations:** ^1^First Clinical Medicine College, Shandong University of Traditional Chinese Medicine, Jinan, China; ^2^Department of Traditional Chinese Medicine, Shandong Provincial Hospital, Jinan, China; ^3^College of Acupuncture and Moxibustion and Tuina, Shandong University of Traditional Chinese Medicine, Jinan, China; ^4^Department of Science and Education, Shandong Academy of Chinese Medicine, Jinan, China; ^5^Department of Gynecology, Laiwu Hospital of Traditional Chinese, Jinan, China; ^6^International Office, Shandong University of Traditional Chinese Medicine, Jinan, China; ^7^Department of Obstetrics and Gynecology, Shenzhen Hospital, Peking University, Shenzhen, China; ^8^Department of Gynecology, Shandong Provincial Hospital, Jinan, China

**Keywords:** neuropathic pain, animal model, neural circuits, brain nuclei, acupuncture

## Abstract

Neuropathic pain (NP) is known to be associated with abnormal changes in specific brain regions, but the complex neural network behind it is vast and complex and lacks a systematic summary. With the help of various animal models of NP, a literature search on NP brain regions and circuits revealed that the related brain nuclei included the periaqueductal gray (PAG), lateral habenula (LHb), medial prefrontal cortex (mPFC), and anterior cingulate cortex (ACC); the related brain circuits included the PAG-LHb and mPFC-ACC. Moreover, acupuncture and injurious information can affect different brain regions and influence brain functions via multiple aspects to play an analgesic role and improve synaptic plasticity by regulating the morphology and structure of brain synapses and the expression of synapse-related proteins; maintain the balance of excitatory and inhibitory neurons by regulating the secretion of glutamate, γ-aminobutyric acid, 5-hydroxytryptamine, and other neurotransmitters and receptors in the brain tissues; inhibit the overactivation of glial cells and reduce the release of pro-inflammatory mediators such as interleukins to reduce neuroinflammation in brain regions; maintain homeostasis of glucose metabolism and regulate the metabolic connections in the brain; and play a role in analgesia through the mediation of signaling pathways and signal transduction molecules. These factors help to deepen the understanding of NP brain circuits and the brain mechanisms of acupuncture analgesia.

## 1. Introduction

In 2008, neuropathic pain (NP) was defined as “pain caused by a lesion or disease of the somatosensory system” by the International Association for the Study of Pain Special Interest Group on Neuropathic Pain, which replaced the 1994 definition. The new definition replaced “dysfunction” with “lesion” or “disease” and “somatosensory system” with “neurological system,” making its situation clearer (Jensen et al., 2011). NP is mainly divided into the peripheral and central categories and can be caused by trigeminal neuralgia, postherpetic neuralgia, peripheral nerve injury, and spinal cord or brain injury (Scholz et al., 2019; Szok et al., 2019). Epidemiological surveys have shown that its prevalence in the general population can range between 6.9 and 10% (Van Hecke et al., 2014), with nociceptive hypersensitivity and abnormal pain being the main features of its development (Cavalli et al., 2019). However, because pain is essentially an unpleasant emotional experience (Raja et al., 2020), the symptoms of NP patients are often accompanied by sleep disturbances, anxiety, depression, and cognitive impairment in addition to the pain itself, and their quality of life is understandably much lower than that of non-NP patients (Attal et al., 2011).

Somatosensory nerves such as mechanoreceptors and injury receptors can send signals to the brain for further processing via the dorsal horn of the spinal cord, and injury or disease of the somatosensory nervous system can lead to altered and disturbed transmission of sensory signals to the brain, resulting in nociception (Colloca et al., 2017). Neuropathic pain is associated with abnormal changes in several specific brain regions (Zhang Y. N. et al., 2022), but the interactions of pain-related neuronal networks are still not fully explored. Therefore, understanding the different brain nuclei and brain neural circuits involved in NP can refine the underlying central neuromodulatory mechanisms of NP.

NP is a major challenge in chronic pain management because of its persistent nature and negative emotions and other symptoms that seriously affect patients’ quality of life. Drugs still dominate the current therapeutic management of NP, but many drugs recommended as first-line treatment offer poor pain relief (Finnerup et al., 2015). The World Health Organization has recommended acupuncture as a non-pharmacologic “first-line therapy” for pain treatment because of its precise analgesic efficacy and high safety (Kelly and Willis, 2019). Although the complex mechanisms underlying acupuncture analgesia remain unclear, there is growing clinical evidence to suggest that acupuncture not only relieves the nociceptive experience of NP but also further modulates the negative emotions and cognitions caused by pain (Paley et al., 2015; Estores et al., 2017; Edwards and Shaw, 2021; Han et al., 2022). Functional brain imaging studies have shown that acupuncture analgesia has region-specific, quantifiable effects on relevant brain structures (Otti and Noll-Hussong, 2012). However, literature on the analgesic effects of NP mediated by acupuncture in specific key brain nuclei or circuits is relatively heterogeneous and fragmented, lacking systematic generalization.

Therefore, we conducted a review of the literature to summarize relevant research on NP animal models and central neuromodulatory mechanisms of brain nuclei and neural circuits associated with NP. We also focus on the key brain nuclei of the nociceptive modulation system and summarize the specific mechanisms of action of acupuncture for NP in recent years. The results showed that acupuncture could exert analgesic effects and regulate pain emotion and cognitive pain effects by affecting functional activities in different brain regions (e.g., synaptic plasticity, the release of different neurotransmitters), which explains the modulatory effects and mechanisms of acupuncture analgesia and further deepens the implication and extension of acupuncture analgesia.

## 2. Animal models of NP and behavioral tests

The etiology of NP is complex, and the construction of different animal models can help understand and explore its pathogenesis and acupuncture treatment mechanisms. Animal models of NP can be classified into spinal cord injury (SCI)-type models, peripheral nerve injury models, chemotherapy-induced NP models, and pain models caused by certain diseases according to the etiology. Specifically, the SCI model includes spinal cord contusion, spinal cord transection, excitotoxicity, and clamped SCI model; the peripheral nerve injury model includes spinal nerve ligation (SNL) model, spared nerve injury (SNI) model, chronic constriction injury (CCI) models, and partial sciatic nerve ligation (PSNL) model; chemotherapy-induced NP models mainly involve the use of vincristine, cisplatin, and paclitaxel; specific disease-induced models mainly include diabetic neuropathic pain model, postherpetic neuralgia model, and the human immunodeficiency virus-induced pain model.

NP includes nociception, emotion, and cognition, so the success of animal model construction is also assessed based on these three dimensions. Furthermore, it mainly relies on the classical behavioral science of mechanical pain, searing pain, and cold pain to evaluate pain, and the specific methods include the von Frey test, hot plate test, cold plate test, and acetone drip test. Negative emotion-related behavioral studies such as depression and anxiety often use the open field test, tail suspension test, forced swim test, and sucrose preference test. Cognitive-related tests include the Morris water maze, novel object recognition, and conditioned place preference tests.

## 3. NP-related brain nuclei and neural circuits

There are about 100 billion neurons in the human brain, and the synaptic connection on neurons can also form a special neural circuit between them to process a variety of complex signals (Luo, 2021). Current studies have found that in the central conduction process of NP, some brain nuclei project onto each other to form different neural circuits that jointly regulate their pain perception, negative emotion, or cognitive impairment.

### 3.1. NP-related brain nuclei

#### 3.1.1. Brain nuclei underlying NP in the brainstem region

The brainstem is a small region between the spinal cord and diencephalon, composed of the medulla oblongata, pons, and midbrain from bottom to top, respectively. The nuclei involved in pain regulation in this region mainly include periaqueductal gray (PAG), rostral ventromedial medulla (RVM), locus coeruleus (LC), ventral tegmental area (VTA), parabranchial nucleus (PBN) and dorsal raphe (DRN).

##### 3.1.1.1. Periaqueductal gray

The central endogenous pain modulatory system is centered on the midline brainstem structures and consists of the descending inhibitory and descending facilitatory systems. The system is mainly composed of the PAG and rostral ventromedial medulla (RVM). The PAG, an important axial structure of the midbrain, comprises four subregions, namely the dorsomedial PAG (dmPAG), dorsolateral PAG (dlPAG), lateral PAG (lPAG), and ventrolateral (vlPAG) sub-regions (Menant et al., 2016). After CCI surgery, vlPAG neurons showed increased excitability (Du et al., 2013). After the onset of behavioral hypersensitivity induced by the chemotherapeutic agent paclitaxel, vlPAG neurons exhibited increased neuronal activity and heat pain-induced changes in neuronal activity. Systemic administration of gabapentin, a non-opioid analgesic, significantly reduced the firing of spontaneous and thermally evoked vlPAG neurons in response to non-injurious and injurious thermal stimuli (Samineni et al., 2017). This is also the leading site of the analgesic effects of opioids or electrical stimulation (Sohn et al., 2000; Lee et al., 2012). These results suggests that the PAG may be an essential target for treating NP.

##### 3.1.1.2. Rostral ventromedial medulla

The RVM also plays an important role in NP regulation. It is an important brain region that integrates downstream pain regulation by pronociceptive on cells and antinociceptive off cells. In SNL rats, neural injury leads to the death of RVM off cells, shifting the downstream control of balance from pain inhibition to pain facilitation (Leong et al., 2011). By injecting lidocaine in the RVM region, the inhibitory neuronal function can be altered, releasing the downstream pain inhibitory system and thus relieving NP (Saadé et al., 2010). The RVM is also essential for maintaining a state of nociceptive sensitization. After nerve injury, both the on and off cells with RVM are sensitive to innocuous and noxious mechanical stimuli, leading to abnormal pain and nociceptive hyperalgesia in the NP state (Carlson et al., 2007).

##### 3.1.1.3. Locus coeruleus

Located in the anterior back of the pons, LC contains the largest norepinephrine (NE) neurons in the central nervous system and is responsible for the internal regulation of pain and emotion. Long-term NP increases LC neuronal activity in both SNI and CCI models (Llorca-Torralba et al., 2019a; Alba-Delgado et al., 2021). Especially in the mouse model of NP combined with depression, it was observed that the expression of Fos protein and spontaneous discharge of LC-NE neurons were significantly increased (Bravo et al., 2014), which suggests that in addition to regulating NP, LC also promoted the development and maintenance of pain-related negative emotions.

##### 3.1.1.4. Ventral tegmental area

The mesolimbic dopamine (DA) reward circuit comprises dopaminergic (DAergic) neurons in the VTA and their projections to several brain regions such as the nucleus accumbens (NAc), prefrontal cortex (PFC), anterior cingulate cortex (ACC), and amygdala, which are critical for nociception (Yang et al., 2020). The VTA is located between the substantia nigra and red nucleus, among 60–65% are dopaminergic neurons, and it is the primary source of dopamine in the midbrain reward system (Beier et al., 2015). For example, VTA-DA neurons have plasticity during chronic NP (Huang S. et al., 2019). In the SCI model, VTA dopaminergic neuron activity is reduced (Ko et al., 2018). However, in a trigeminal neuralgia (TN) model, the activity of VTA DA neurons was increased (Zhang L. et al., 2021), and gabapentin was shown to reverse depression-like behavior in painful states by modulating the VTA-DA neurons (Fu et al., 2018). The above results suggest that VTA-DA neurons have a bidirectional regulatory effect on NP.

##### 3.1.1.5. Parabranchial nucleus

The PBN is located in the dorsolateral part of the pons and is divided into three main subnuclei, namely the medial parabrachial nucleus, the lateral parabrachial nucleus (LPB), and the ventral Kölliker-Fuse nucleus. The PBN is a key regulator of NP (Wang and Xu, 2021). Significantly increased activity of the PBN was observed in TN or SCI rat models, and inhibition of PBN neurons relieved abnormal pain and nociceptive hyperalgesia (Uddin et al., 2018; Li J. et al., 2023). The PBN contains mostly excitatory glutamatergic (Gluergic) neurons and a small number of inhibitory γ-aminobutyric acid (GABA) neurons. Furthermore, direct synaptic connections exist between the two neurons, and local inhibitory neurons can suppress excitatory neuronal activity. In the NP model, Glu neurons were heavily activated in the LPB. Further activation of excitatory neurons by optogenetic techniques significantly caused mechanical nociceptive hypersensitivity, whereas inhibition of excitatory neurons in this brain region effectively relieved pain symptoms. Although only about 10% of GABAergic neurons were activated, activating inhibitory neurons also alleviated pain (Sun et al., 2020). These results suggest that both Glu and GABAergic neurons in the LPB brain region encode critical information for NP. Moreover, at the microcircuit level, a delicate balance between LPB excitatory and inhibitory neuronal activity determines the development and maintenance of NP.

##### 3.1.1.6. Dorsal raphe

Co-morbid depressive symptoms in chronic pain disorders are a prevalent health problem, and research on depression has focused on 5-hydroxytryptamine (5-HT) and 5-HT ergic neurotransmission for many years (Dell’Osso et al., 2016). The DRN is an important pain-modulating nucleus rich in 5-HT neurons and many other neurotransmitters containing neuronal neuromodulators (Wang and Nakai, 1994). 5-HT neurons have pain inhibitory effects, and one study found that EA treatment reduced pain-depression symptoms in rats may be related to the upregulation of 5-HT expression in the DRN (Wu et al., 2017).

#### 3.1.2. Brain nuclei underlying NP in the diencephalon region

The diencephalon is located between the brainstem and the cerebrum, connecting the cerebral hemispheres to the midbrain. It is generally divided into five parts: the dorsal thalamus, posterior thalamus, superior thalamus, basal thalamus, and hypothalamus. The nuclei involved in the regulation of NP include Lateral habenula (LHb), paraventricular nucleus of the thalamus (PVT).

##### 3.1.2.1. Lateral habenula

The habenula is an epithalamic structure of the limbic system and is divided into two parts: the lateral habenula (LHb) and medial habenula (MHb). In particular, the LHb plays a crucial role in pain and depression anxiety behaviors. A recent study reported that LHb Glu is activated after infraorbital nerve injury and selective inhibition of these neurons relieved postoperative anxiety but not abnormal orofacial pain (Cui et al., 2020). Similarly, cerebral blood flow in the LHb is increased (Paulson et al., 2000), LHb neurons are overactivated after sciatic nerve injury, and selective inhibition of LHb Glu relieves mechanical abnormalities in pain and negative emotions caused by nerve damage, suggesting that LHb Glu plays a vital role in NP and pain affection (Du et al., 2021).

##### 3.1.2.2. Paraventricular nucleus of the thalamus

The PVT belongs to a group of midline thalamic nuclei composed mainly of Gluergic neurons and is associated with many emotional triggers such as aversion, fear (Zhu Y. B. et al., 2022), comfort, and anxiety (Zeng et al., 2021). Recent studies have detected hyperexcitability of PVT neurons in a mouse model of NP and inflammation. Injury or inhibition of PVT neurons attenuated abnormal pain and nociceptive hyperalgesia in mice (Chang et al., 2019; Zhang W. T. et al., 2022). Similarly, one study showed significant relief of visceral pain caused by acute pancreatitis after damaging the PVT by using the DREADDs technique (Urban and Roth, 2015), which suggests that the PVT plays an important role in the modulation of multiple pains such as visceral pain, inflammatory pain, and NP.

#### 3.1.3. Brain nuclei underlying NP in the brain region

The cerebral hemispheres are covered with gray matter, called the cerebral cortex, and their deeper side is composed of white matter, called the medulla, with gray matter nuclei within the medulla as the basal ganglia. The nuclei involved in pain regulation mainly include medial prefrontal cortex (mPFC), the anterior cingulate cortex (ACC), amygdala and hippocampus.

##### 3.1.3.1. Medial prefrontal cortex

The PFC is the cortical area covering the anterior frontal lobe and receives input from all other cortical areas and functions to guide motor, cognitive, emotional, and social behavior (Kolb et al., 2012). According to its anatomical location, the PFC can be divided into four parts: dorsolateral PFC, dorsomedial PFC (dmPFC), ventral medial PFC (vmPFC), and orbitofrontal cortex. Among them, mPFC is composed of the ACC, prelimbic (PL), and infralimbic cortex, and is a key part of the cortical representation of pain. In the SNI-induced NP rat model, the mPFC mesopotentials are significantly increased (Sang et al., 2018) and morphological and functional abnormalities are present (Metz et al., 2009).

##### 3.1.3.2. Anterior cingulate cortex

The ACC is a prefrontal area involved in processing painful emotions and encoding emotional information. Its pyramidal neurons show hyperactivity in NP conditions (Zhao et al., 2018). In NP mice, the firing rate of pyramidal cells but not inhibitory interneurons in the ACC is increased, suggesting that increased pyramidal cell activity allows for an impaired excitatory/inhibitory balance in the ACC and exacerbates pain hypersensitivity in mice with NP (Zhu D. Y. et al., 2022). The ACC is also a key center for pain-induced depression (Barthas et al., 2015). When anxiety-depression-like consequences occur in neurologically injured mice, the firing rate within the ACC is significantly increased. Optogenetic inhibition of its hyperexcitable state is sufficient to alleviate the aversive and anxiety-depression-like consequences of NP (Sellmeijer et al., 2018). Microinjection of oxytocin into the ACC attenuates NP animals’ detrimental and anxiety-like responses by enhancing inhibitory transmission and stimulating ACC interneurons’ behavioral responses (Li et al., 2021). These suggest that hyperactivity of the anterior cingulate cortex is critical for driving NP and negative emotions.

##### 3.1.3.3. Amygdala

The amygdala contains several subregions and nuclei, including on the lateral side the BLA and CeA on the lateral side connected in series (Swanson and Petrovich, 1998). The plasticity of the amygdala plays an important role in emotional pain behavior and its regulation through affective states (Neugebauer et al., 2004). It has been observed that the volume of the amygdala increases in SNI-induced NP rats (Gonçalves et al., 2008). In a mouse model of paclitaxel-induced NP, there were significantly more c-Fos-positive neurons in BLA and CeA than in control mice. Selective inhibition of CaMKII neurons in BLA alleviated anxiety behaviors (Liu et al., 2022). CeA neurons can be sensitized by reducing GABAergic inhibition, which contributes to developing anxiety-like behaviors associated with neuropathic pain in rats (Jiang et al., 2014). The above studies provide ample evidence that the amygdala plays a crucial role in the pathogenesis of NP and associated negative emotions.

##### 3.1.3.4. Hippocampus

The hippocampus is an essential component of the limbic system of the brain, and it is the main body of the hippocampus, composed of CA3, CA2, and CA1 regions. It is the center of learning, memory, and emotional responses. More and more evidence suggests that the hippocampus is involved in forming and maintaining neuropathic pain. Excitatory amino acids play an important role in the hippocampus during neuropathic pain. Mechanical hypersensitivity and recognition defects were observed in rats with peripheral nerve injury, with a significant decrease in hippocampal GLu concentration and reduced excitatory synaptic transmission (Xiong et al., 2020). Dopamine plays a vital role in synaptic plasticity in the hippocampus, and the characteristics of working memory impairment in SNI animal models depend on hippocampal dopaminergic signals (Cardoso-Cruz et al., 2014). In addition, the activation of hippocampal microglia and subsequent pro-inflammatory responses play a crucial role in developing neuropathic pain and the pathogenesis of anxiety and depression. CCI can cause mechanical and thermal hyperalgesia, regulating the activation of microglia and subsequent polarization of pro-inflammatory phenotypes, leading to the pathogenesis of neuropathic pain. Antibiotic administration reversed these abnormalities, suggesting that it may be a new method for treating neuropathic pain (Zhou et al., 2021). In the TN model, the activation of unilateral hippocampal microglia directly leads to anxiety and depression and damage to hippocampal long-duration potentiation (LTP), accompanied by upregulation of hippocampal inflammatory factor levels (Chen et al., 2023). Neurological damage can lead to dysfunction of hippocampal neurons and glial cells. Neuropathic pain can be improved by regulating the expression level of hippocampal inflammatory factors.

### 3.2. NP-related brain neural circuits

#### 3.2.1. PAG-RVM

The PAG starts the endogenous pain modulatory system and is densely projected to the RVM. The vlPAG sends glutamatergic (Gluergic) projections to the RVM, and persistent activation of metabotropic glutamate (GLu) receptors in the PAG can increase pain thresholds and suppress chronic NP by modulating the activity of RVM OFF cells (Chung et al., 2020; Hosseini et al., 2020). Eliminating the projection from vlPAG to RVM can lead to the generation of NP (Huang J. et al., 2019). Another study showed that PAG neurons project anatomically and functionally to the RVM and exert a descending facilitatory effect on mice with NP. In a chemotherapy-induced NP model, somatostatin neurons from PAG to RVM facilitate NP. Inhibition of PAG-RVM projections attenuates the mechanical and thermal hypersensitivity responses associated with neuropathy (Zhang et al., 2023). The above studies suggest that the PAG-RVM neural circuit can exert the descending inhibitory and facilitatory pathways to regulate the development of NP in a bidirectional manner, respectively.

#### 3.2.2. LC-PFC

In addition to the well-known descending locus coeruleus to spinal cord (LC-SC) pain control pathway (Li J. et al., 2022), the ascending pathway through this nucleus may be responsible for moving to the higher center of pain processing (Llorca-Torralba et al., 2016). In an NP model, LC-SC activation reduced hindlimb sensitivity and induced conditioned place preference. In contrast, activating LC-PFC exacerbated spontaneous pain, generated aversion, and increased anxiety-like behavior (Hirschberg et al., 2017). The LC-NE system also plays a vital role in sleep–wake regulation (Van Egroo et al., 2022). NP is thought to severely interfere with sleep. Sciatic nerve ligation produced significant thermal hyperalgesia and sleep disturbance. The release of NE levels in the PFC is significantly increased by stimulating LC neurons (Koh et al., 2015), which suggests that hyperactivation of LC-PFC neurons may be related to sleep disturbance under NP.

#### 3.2.3. LC-BLA

In the CCI rat model, LC neurons can project to the basolateral amygdala (BLA). Chemogenetic blockade of the LC-BLA circuit abolished long-term pain-induced anxiety and fear but did not change the perception or episodic memory (Llorca-Torralba et al., 2019b). These results showed that the LC-BLA pathway is overactivated in the comorbidity of chronic pain and anxiety, whereas other pathways may independently process the cognitive dimension related to pain.

#### 3.2.4. VTA-NAc

The DA neuronal projections from the VTA to the NAc are a central part of the midbrain reward circuit and have been the focus of scientists studying the neural circuit of pain. However, in recent years, the understanding of the involvement of the VTA-NAc DA circuit in the modulation of pain sensation has varied considerably among different studies.

In an NP model mouse, the excitability of VTA-DA neurons projecting to the NAc is significantly reduced, and optogenetic stimulation of VTA-DA neurons significantly relieved pain (Watanabe et al., 2018). In contrast, in several other studies, elevated c-Fos expression was observed in the VTA-NAc DA neurons of CCI mice. Optogenetic inhibition of dopamine release from the VTA to the NAc reversed CCI-induced thermal nociceptive hyperalgesia (Zhang et al., 2017; Wang et al., 2021).

In some cases, in addition to DA, the VTA releases GABA or Glu neurons, which mediates pain and related behaviors (Zell et al., 2020; Cai and Tong, 2022). In the CCI pain model, VTA Gluergic neurons’ activity was significantly increased and projected to the NAc. Inhibition of Glu projections from the VTA to the NAc enhanced pain tolerance and alleviated pain-induced anxiety and depression (Abdul et al., 2022). Increased activity of GABAergic neurons in the VTA, in turn, alleviated SCI-induced pain and negative emotions and motivation by inhibiting DA neuronal activity (Ko et al., 2018). The above results indicate heterogeneity in the involvement of VTA neurons or VTA-NAc circuits in pain sensation modulation.

#### 3.2.5. Lateral hypothalamus-VTA-NAc

In addition, the VTA can also receive GABAergic projections from the LH and modulate pain perception by targeting local GABAergic neurons to disinhibit dopamine. In the CCI mouse model, LH GABA neuronal projections to VTA-DA neurons consistently inhibit GABA neuronal activity in the VTA region, i.e., indirectly inhibiting inhibitory neuronal activity in the VTA region via the LH-VTA circuit, thereby increasing DA neuronal activity, increasing dopamine release in the NAc, and ultimately inducing pain (Ma et al., 2023).

#### 3.2.6. LPB-CeA

Most GABAergic input to the LPB is derived from and innervated by CeA (Torres-Rodriguez et al., 2023). In the NP model, enhanced activity of LPB neurons is associated with a decrease in inhibitory GABAergic input, and activation of this pathway inhibits pain behavior (Raver et al., 2020). In addition, LPB neurons can also transmit nociceptive information (Liang et al., 2016) and negative emotions (Qiao et al., 2019) to CeA. In the SNL model, LPB-CeA potentials significantly increase and manifest as abnormal tactile pain (Nakao et al., 2012). Specific activation of this circuit can exacerbate pain and behavioral problems such as sleep disorders and anorexia nervosa (Katagiri and Kato, 2020). It follows that at the brain circuit level, LPB and CeA are regulated bidirectionally, jointly mediating either upstream injurious signaling or downstream pain modulatory information.

#### 3.2.7. LPB-VTA

VTA is another downstream projection target of LPB. In chronic NP combined with depression states, LPB receives Glu-ergic neurons from the trigeminal spinal caudate subnucleus (Sp5C) and its projections to the VTA, leading to increased firing activity of VTA-DA neurons. Inhibition of this pathway reverses depressive-like behavior, thus “decoupling” chronic pain from co-morbid depression (Zhang L. et al., 2021). This finding suggests that although depressive-like behaviors are caused by NP, depression and pain may be processed via separate controls in the brain.

#### 3.2.8. LPB-SNR-VTA-NAc

Some PBN neurons can receive injury-receptive signals upstream from the spinal cord and transmit them to the substantia nigra pars reticulata (SNR) of the midbrain and can be activated by mechanical and thermal pain stimuli. In SNL pain models, optogenetic inhibition or specific ablation of this pathway can block pain responses (Yang et al., 2021), suggesting that this neural circuit transmits pain information and that inhibition of this circuit may exert some analgesic effect.

However, information transmitted from the LPB to SNR not only forms direct inhibitory synaptic connections with VTA neurons via GABA-ergic neurons but also inhibits the spontaneous firing of LPB-VTA neurons, thus relieving the latter from excitatory effects on VTA-DA neurons (Yang et al., 2021). The two pathways are structurally different but exhibit inhibitory effects on VTA-DA neurons. The SNR receiving LPB projections innervates VTA-DA neurons through direct and indirect pathways to induce pain, further elucidating the neural mechanisms underlying the regulation of pain signal transmission by LPB-SNR and LPB-VTA circuits.

#### 3.2.9. LHb-DRN

As a key target of pain modulation, LHb also sends outputs to the dorsal raphe nucleus (DRN) to modulate pain and depression-like behaviors. Among these, the 5-HTergic system of the nucleus accumbens is an important component of pain processing and depression pathogenesis. In a CCI model, LHb neuronal activity was increased, and DRN neuronal activity and 5-HIAA/5-HT ratio were decreased, accompanied by depression-like behavior. lHb lesions improved pain thresholds and depression-like behavior in CCI model rats by increasing the 5-HT levels in the DRN. The results suggest that increased LHb-DRN pathway activity explains the coexistence of pain and depression (Li et al., 2017).

#### 3.2.10. LH-LHb

The LH is located in the lateral region of the hypothalamus. Much of the evidence for the function of LH has been focused on the control of feeding behavior, but fewer studies have focused on its role in pain regulation (Devarakonda and Kenny, 2017; Yonemochi et al., 2019). lHb is one of the major projection areas of the LH brain region that respond to injurious stimuli. Recent studies have found that the LH-LHb neural circuit involves the central mechanisms regulating NP. Using brain slice membrane clamp, chemical genetics and transgenic techniques, a research team found that CCI induced increased activity of Gluergic neurons and LH-LHb excitatory neural circuits in the LH brain region in a mouse model of NP. Inhibition of LH Gluergic neurons or LH-LHb circuits relieved NP, while specific activation decreased the mechanical retraction threshold and thermal retraction latency (Gu et al., 2023), suggesting that the projections sent by LH glutamatergic neurons to LHb play a vital role in the regulation of NP and that the LH-LHb circuit may be a new target for the prevention and treatment of NP.

#### 3.2.11. PVT-CeA-PAG

In SNI rats, glutamatergic excitatory neurons within the PVT were observed to send projections to CeA by using the neuroanatomical tract-tracing techniques and acted on GABAergic inhibitory neurons within this nucleus; the latter, in turn, acted downstream on glutamatergic neurons within the vlPAG to facilitate NP signaling (Liang S. H. et al., 2020). In conclusion, the PVT-CeA-PAG pathway facilitates NP messaging, and activation of this pathway can decrease mechanical pain thresholds in normal animals; therefore, inhibition of this circuit can effectively exert analgesic effects in chronic pain states.

#### 3.2.12. PVT-vmPFC

The vmPFC is a key node in cortical and subcortical networks and plays a critical role in altering the meaning of pain (López-Solà et al., 2018). It was found that the vmPFC receives Gluergic projections from the PVT and drives the activation of vmPFC-nNOS-expressing neurons to convert pain signals into anxiety signals (Liang H. Y. et al., 2020). These elucidated the modulatory role of the PVT-PFC neural circuit on chronic NP-induced anxiety, which may become a new target for pain-induced anxiety intervention therapy. As the circuit is not associated with nociception, it may be related to different types of neurons transmitted.

#### 3.2.13. mPFC-NAc

DA is imported from the VTA to the mPFC and modulates NP-related behaviors in mice (Huang et al., 2020). Moreover, a key output target of the PFC is the NAc. In a rat model of persistent NP, optogenetic activation of the PFC produces substantial analgesic effects and reduced affective symptoms of pain. Projections to the NAc likely mediate this analgesic function (Lee et al., 2015).

#### 3.2.14. dmPFC-vlPAG

Previous studies have focused on the PAG-RVM downstream pain modulation circuit. In fact, mPFC can also exert important top-down control over pain perception by projecting directly to the PAG. Using multiple tracing methods, one study team identified an excitatory downstream neural pathway from the dmPFC to the vlPAG. In a mouse model of chronic NP, analgesic and anxiolytic effects were produced by specific activation of the dmPFC-vlPAG circuit, while silencing or inhibiting this circuit promoted chronic pain and anxiety-like behaviors (Yin et al., 2020). In line with the results of another study, PAG was found to receive Gluergic projections from mPFC, and activation of these Gluergic projections in vlPAG inhibited mechanical and thermal nociceptive hyperalgesia in the ipsilateral paw of SNI mice (Huang J. et al., 2019).

#### 3.2.15. PL-ACC

The mPFC receives input from subcortical areas and integrates relevant information for transmission to other brain regions, such as the PAG and the NAc. In addition, the mPFC also projects extensively to other cortical layers such as the ACC. In a mouse model of peripheral nerve injury-induced NP, the activity of neurons in the PL that project to the ACC is significantly reduced. Chemical genetics activate the PL-ACC circuit and can attenuate pain-like behavior in mice (Li M. et al., 2022).

#### 3.2.16. ACC-NAc/VTA

NAc and VTA are two key nodes of the limbic dopamine system in the midbrain. One study reported that the ACC projects D2 medium spiny neurons and GABAergic neurons to the NAc and VTA, respectively, to regulate aversive emotions in NP. Chemical genetics inhibition of these projections induced conditioned location preference without modulating evoked pain thresholds (Gao et al., 2020). In conclusion, the findings suggest that projections from the ACC to the NAc and VTA mediate NP-related aversive behavior.

#### 3.2.17. rACC-vlPAG

The PAG also receives fiber projections from the ACC. Activation of Gluergic projections from the rostral ACC (rACC) to the vlPAG induced nociceptive hypersensitivity and anxiety-like behaviors. Inhibition of this circuit attenuated pain and anxiety behavior in the SNI model (Zhu et al., 2021). This study reveals a novel circuit, the rACC-vlPAG pathway, and mediates NP and pain-induced anxiety.

#### 3.2.18. BLA-mPFC-PAG

Peripheral nerve injury alters the synaptic input from BLA to mPFC, and inhibition of BLA-mPFC projection produces analgesia. Subsequently, it was further determined that mPFC Gluergic neurons project to the PAG and innervate CaMKIIα-positive projection neurons and GABAergic neurons in the vlPAG. Activation of Gluergic projections in the vlPAG inhibited mechanical and thermal nociceptive sensitization in the ipsilateral paw of SNI mice, and inhibition of the vlPAG decreased the ipsilateral mechanical threshold of SNI mice, thereby exacerbating NP. Enhanced feed-forward inhibition of PN in the mPFC reduced the activity of mPFC-vlPAG projections and exacerbated the pain response, while facilitating the firing of these projecting neurons via optogenetics had an analgesic effect (Huang J. et al., 2019). In conclusion, nerve injury enhances synaptic inputs to inhibitory interneurons from the BLA to the mPFC, and these enhanced synaptic connections mediate the feed-forward inhibition of projections from the PFC to the mPAG region and its downstream targets, suggesting that mPFC-vlPAG projections are an integral downstream pathway of the BLA-mPFC to control pain responses.

Projections from the BLA to mPFC are also critical for the encoding, transferring, and storing of remote fear memories. Persistent NP significantly affects consolidated fear memory, and the underlying mechanism is likely to be increased inhibitory input to BLA to mPFC projection neurons (Cardenas et al., 2019). Increased fear responses to consolidated fear memories often predict the later development of anxiety and depressive symptoms associated with chronic pain. However, whether BLA-mPFC brain circuits are involved in NP-related negative emotions remain to be further investigated.

**Figure 1:**
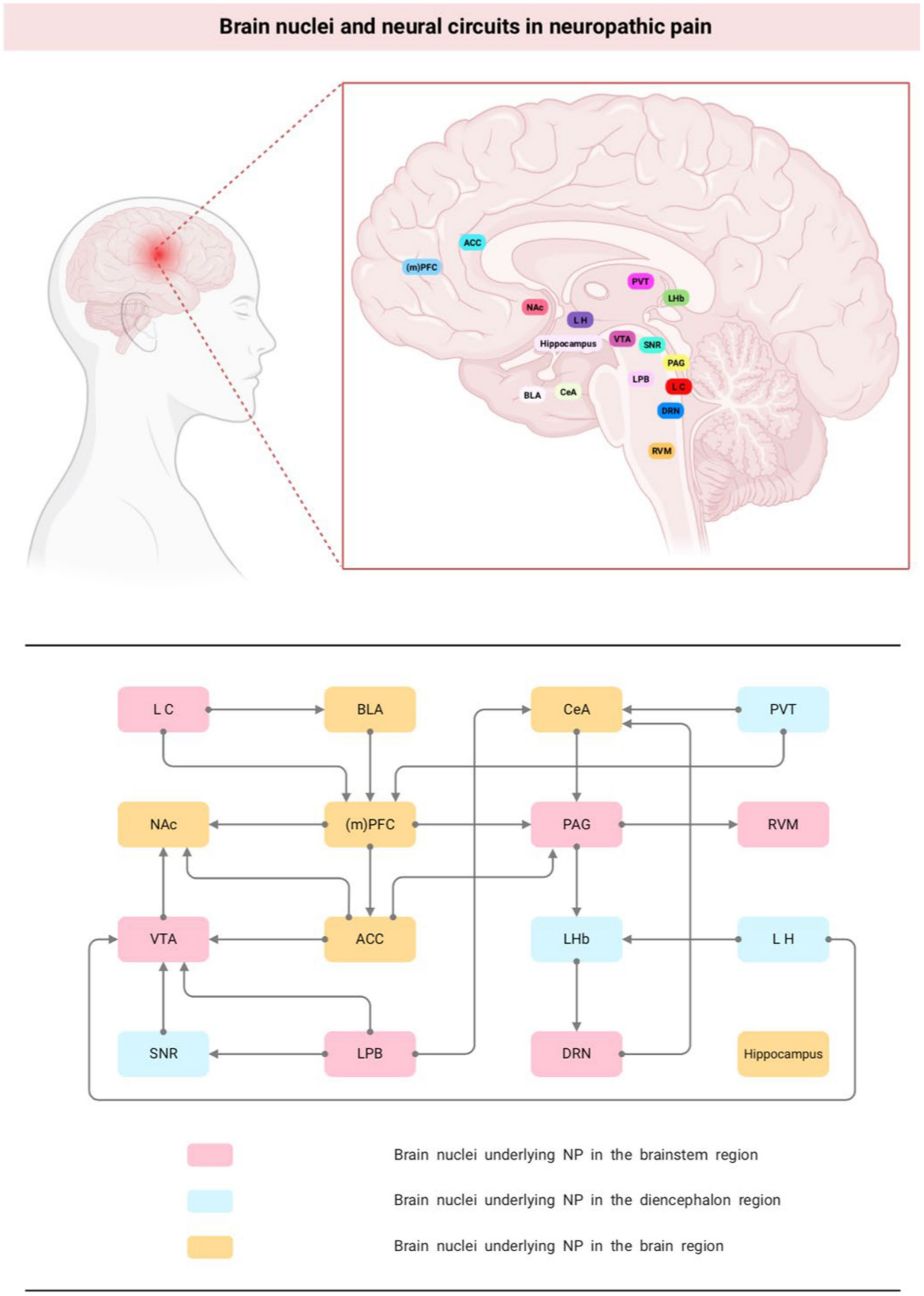
Brain nuclei and neural circuits in neuropathic pain. NP, neuropathic pain; (m)PFC, medial prefrontal cortex; NAc, nucleus accumbens; PAG, periaqueductal gray; ACC, anterior cingulate cortex; VTA, ventral tegmental area; DRN, dorsal raphe nucleus; CeA, central nucleus of the amygdala; LHb, lateral habenula; BLA, basolateral amygdala.

The brain nuclei and neural circuits behind NP are complex and large. Currently, it has been found that nuclei involved in NP nociceptive modulation exist in almost all brain regions, such as PAG and RVM in the brainstem region; LH and PVT in the mesencephalon; and the amygdala, ACC, and PFC in the brain. In addition, some of the nuclei project networks to each other, such as PAG-RVM, LH-LHb, LPB-VTA, PL-ACC, and when one of the links in the feedback-regulated neural circuit formed by them is abnormal, the overall pain modulation equilibrium is altered, which leads to the occurrence of pain (Figure 1). Moreover, owing to the heterogeneity of neuron types distributed between different brain nuclei, such as excitatory Gluergic neurons and inhibitory GABA neurons, different or even opposite behavioral outcomes often occur in the same nucleus or circuits. Therefore, clarifying the supraspinal nuclei and nerve conduction loops associated with NP and analyzing the changes in neuronal activity can help to further understand the pathophysiological mechanisms of pain and provide theoretical guidance for the clinical prevention and treatment of NP and the associated emotional or cognitive disorders induced by pain.

## 4. Brain mechanisms involved in the treatment of NP by using acupuncture

Acupuncture analgesia results from the interaction in the central nervous system between nociceptive afferent signals and afferent signals from acupuncture points. Current research has shown that many critical nuclei in the brainstem, mesencephalon, cerebrum, and other brain regions, such as PAG, ACC, hippocampus, and amygdala, are involved in the transmission process of harmful information and that the interconnection of each other’s network links further regulates the development of NP. At the brain level, acupuncture can inhibit the conduction of harmful stimuli by directly affecting the functional activities of these brain nuclei or neural circuits, thereby improving pain. For example, enhanced neural activity was observed in the primary somatosensory cortex (S1) of nerve-injured rats, and optical signals and region of activation in S1 were significantly reduced after electroacupuncture (EA) stimulation. This indicates that EA stimulation inhibits excitatory nerve signals in the S1 cortex induced by noxious stimuli in NP (Cha et al., 2017). A dynamic causal modeling study found that harmful stimuli are likely driven by the somatosensory cortex, propagating bidirectionally with the hypothalamus and amygdala, as well as their interactions. In contrast, needling reduced limbic–cortical feedback in a rat model of NP (Ma et al., 2020). Functional magnetic resonance imaging (fMRI) of the resting neural network showed reduced dlPFC and ACC activity in patients with chronic sciatica. After acupuncture treatment, activity in these areas was primarily normalized (Li et al., 2012). Taken together, this suggests that many brain nuclei are common information integration centers for afferents from needling and other harmful stimuli.

**Figure 2:**
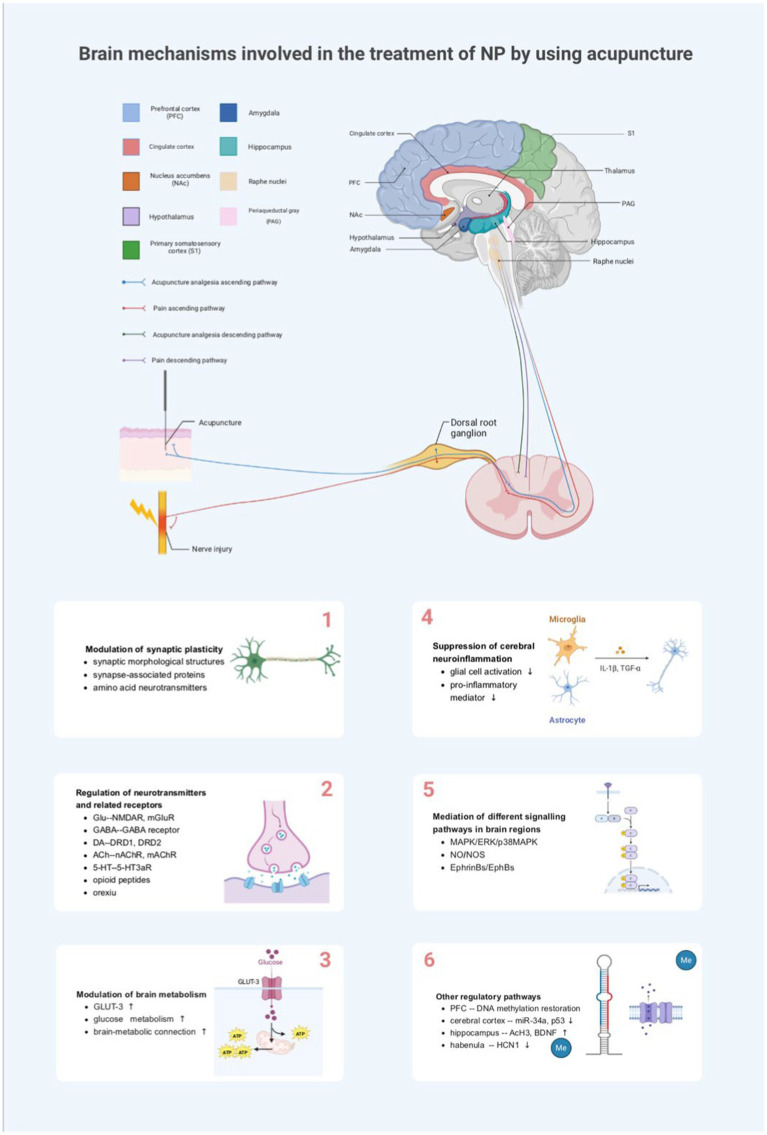
Brain mechanisms involved in the treatment of NP by using acupuncture. NP, neuropathic pain; PFC, medial prefrontal cortex; NAc, nucleus accumbens; PAG, periaqueductal gray; S1, primary somatosensory cortex; TNF-α, tumor necrosis factor-alpha; IL-1 β, interleukin-1beta; Glu, glutamate; NMDA-R, N-methyl-D-aspartate receptor; mGluR, metabotropic glutamate receptor; GABA, γ-aminobutyric acid; 5-HT, 5-hydroxytryptamine; 5-HT3aR, 5-HT3a receptor; DRD1, dopamine receptor D1; DRD2, dopamine receptor D2; nAChR, nicotinic acetylcholine receptor; mAChR, muscarinic acetylcholine receptor; GLUT3, glucose transporter 3; ATP, adenosine triphosphate; MAPK, mitogen-activated protein kinase; ERK, extracellular-regulated protein kinase; p38MAPK, P38 mitogen-activated protein kinase; NO, nitric oxide; NOS, nitric oxide synthase; Me, methylation; BDNF, brain-derived neurotrophic factor; AcH3, acetylated histone H3; HCN1, hyperpolarization-activated cyclic nucleotide-gated channels 1.

However, relevant neuronal synapses, neurotransmitters, glial cells, and other biologically active substances in the brain are altered during the generation and transmission of pain. Acupuncture relieves NP by affecting synaptic plasticity in various brain regions; facilitating the release of neurotransmitters, inflammatory mediators, neuropeptides, and other substances; regulating glucose metabolism in brain regions; and/or mediating specific pain-related signaling pathways (Figure 2).

### 4.1. Acupuncture modulates synaptic plasticity in the treatment of NP

Synapses are the central hub of information transmission between neurons and have plasticity, i.e., changes in synaptic structure and transmission efficacy result in adaptive changes in neural circuits. It has been shown that sustained harmful stimuli can cause spinal or brain synapses to increase in strength and efficacy dependently and that these plastic changes contribute to NP hypersensitivity (Costigan et al., 2009; Latremoliere and Woolf, 2009). The human hippocampus is highly plastic for learning and memory acquisition (Neves et al., 2008). For example, SNI induces LTP of C-fiber synapses in the dorsal horn of the spinal cord but impairs LTP in the hippocampus. Opposite changes may lead to NP and memory deficits, respectively (Liu et al., 2017). The amygdala, ACC, and other key brain nuclei for sensing emotion-related behaviors such as pain and anxiety are also highly plastic (Zhuo, 2020; Ge et al., 2022). Thus, modulating structural or functional plasticity in brain synapses can inhibit central sensitization and thus reduce NP. Pain relief induced by repeated acupuncture is often accompanied by improved synaptic plasticity in brain nerves. Electroacupuncture stimulation attenuates abnormal orofacial pain and anxiety-like behaviors in a TN mouse model by modulating synaptic plasticity in hippocampal CA1 neurons (Jia et al., 2022). Repeated EA of bilateral ST36 (USANi) and GB34 (YangLingQuan) had a cumulative analgesic effect, which was mainly related to remodeling of the synaptic structure (synaptic gap width and postsynaptic density) in the paraventricular nucleus of the hypothalamus (Xu et al., 2013).

Acupuncture can also modulate synaptic plasticity by relying on the regulation of synapse-associated proteins such as calmodulin-dependent protein kinase II (CaMKII), postsynaptic density protein-95 (PSD-95), and synapsin, among others. For example, the cumulative analgesic effect of repeated EA also involves synaptic remodeling in the hippocampal CA3 region, which was mainly associated with the upregulation of calcium/calmodulin-dependent protein kinase II expression in the hippocampal CA3 region (Xu et al., 2012). Acupuncture treatment significantly improved abnormal pain and pain-induced cognitive dysfunction in PSNL mice. In addition, acupuncture enhanced the expression of Ca^2+^/CaMK II and synaptic proteins (pPSD-95 and pSyn-1). These results suggest that acupuncture can modulate synaptic efficacy by enhancing the expression of synapse-related proteins in the hippocampus, thereby improving NP-associated pain and cognitive function (Jang et al., 2019). Yin et al. (2023) found that acupuncture can improve cognitive impairment by mediating hippocampal synapses’ structural and functional plasticity. The number and density of neuronal dendritic spines in the CA1 region of the hippocampus and synaptophysin in the hippocampal tissues of the CCI model were significantly lower than those of normal mice. By contrast, acupuncture could improve the social and cognitive behaviors of mice experiencing chronic pain by modulating the expression of hippocampal synaptophysin and changes in the dendritic spines of neurons (Yin et al., 2023). Other studies have shown that repetitive EA relieves pain sensation and pain mood components in rats with chronic neuropathic pain, which may also be related to the up-regulation of the PSD-95, metabotropic Glu receptor, and GABA receptor subtypes protein expression in the amygdala, and the improvement of synaptic plasticity (Feng et al., 2014; Duanmu et al., 2017).

The above animal experiments suggest that improving synaptic plasticity in different brain regions is one of the mechanisms by which acupuncture relieves NP, especially in the hippocampus, paraventricular nucleus of the hypothalamus, and amygdala. On the one hand, acupuncture can regulate synaptic morphological structures, such as the number and density of neuronal dendritic spines and synaptic gap width. On the other hand, acupuncture can repair synaptic functional plasticity by regulating the expression of synapse-associated proteins such as CaMKII and PSD-95, as well as the levels of amino acid neurotransmitters. The two ultimately mediate improving symptoms such as NP, depression, anxiety, negative mood, and cognitive memory dysfunction.

### 4.2. Acupuncture modulates neurotransmitters and receptors in the treatment of neuropathic pain

Neurotransmitters form the material basis for the generation of acupuncture effects. Disruption of the balance of excitatory and inhibitory neurotransmitters in different brain regions can lead to chronic NP, so targeting neurotransmitters and related receptors may be one of the potential mechanisms of acupuncture for NP. Moreover, there are differences in the neurotransmitters released from different brain regions involved in acupuncture analgesia, such as glutamate, γ-aminobutyric acid, dopamine, and acetylcholine.

#### 4.2.1. Amino acid neurotransmitters

Glutamate is the most common excitatory neurotransmitter in the central nervous system. EA may exert analgesic effects in NP by reducing glutamate release. In a rat model of CCI, EA significantly relieved nociceptive hypersensitivity and was accompanied by a decrease in glutamate levels in the hippocampus (Huang C. P. et al., 2019). Gluergic projections activating rACC-vlPAG are known to induce nociceptive hypersensitivity and anxiety-like behavior. EA (ST36, SP6) alleviated symptoms of anxiety-like behavior and nociceptive hypersensitivity in SNI mice by inhibiting Gluergic projections in the rACC-vlPAG pathway. By contrast, chemical genetics activation effectively blocked the analgesic effect of EA in the SNI mouse model (Zhu et al., 2021).

The expression of glutamate receptors in different brain regions also plays an important role in the EA treatment of NP pain mood and pain perception, mainly including N-methyl-D-aspartate receptor (NMDAR) and metabotropic glutamate receptor (mGluR). In the hippocampus of CCI rats, the phosphorylation of NMDAR subunit type 1 (pNR1) level was reduced. Continuous EA treatment significantly improved the behavioral deficits in CCI rats and effectively blocked the decrease in pNR1 levels. These results suggest that EA has antidepressant and anxiolytic effects in rats with neuropathic pain, which may be related to the restoration of hippocampal pNR1 (Li et al., 2014). In the PSNL mouse model, EA restored reduced excitatory postsynaptic potentials and increased the hippocampus’s NR2B and GluR1 expression levels. This suggests that acupuncture can ameliorate NP and induce cognitive deficits by modulating hippocampal glutamate receptors (Jang et al., 2019). Similarly, another study reported that repetitive EA on ZuSanLi and YangLingQuan could significantly improve the pain affective component in chronic neuralgia rats, and its effect might be related to the up-regulation of the expression of GluA 1 protein in the amygdala of rats (Yan et al., 2016).

GABA is an essential class of inhibitory neurotransmitters that exert their biological effects mainly by binding to specific GABA receptors. It was found that 15 Hz EA relieved CCI-induced neuropathic pain behavior and increased GABA receptors in the hippocampus and PAG. Moreover, GABA was raised in the PAG upon EA treatment, which suggests that EA treats NP by upregulating the expression of inhibitory neurotransmitters and receptors in the hippocampus and PAG (Huang C. P. et al., 2019). In addition, EA may modulate the excitatory-inhibitory balance of the S1 local circuits to alleviate NP, including the enhancement of GABAergic growth inhibitory hormone-positive interneurons and the inhibition of excitatory pyramidal neurons and vasoactive intestinal peptide-positive interneurons. In contrast, EA-mediated pain hypersensitivity relief and cortical modulation are dependent on the activation of endogenous cannabinoid receptor 1 (CB1) (Wei et al., 2021).

CB1 receptors are widely expressed in nerve endings of GABAergic and Gluergic neurons in the PAG, and activation of CB1 receptors may modulate both GABAergic and Gluergic neurotransmission. In contrast, EA can exert analgesic effects by simultaneously inhibiting GABAergic neurons and stimulating Gluergic neurons via CB1 receptors. Specific knockdown of CB1 receptors attenuates or eliminates some of the effects of EA on pain hypersensitivity (Zhu et al., 2019). In addition, it has also been reported to observe that repeated EA stimulation modulates both amygdala Gluergic NMDAR subunit and GABA receptor subunit expression in a CCI model, which together acts to reduce excitatory synaptic transmission for analgesia (Feng et al., 2014; Duanmu et al., 2017). Thus, EA works together to exert analgesic effects in NP by regulating the expression of Gluergic and/or GABAergic neurons and related receptors and maintaining the relative balance between the two, which also provides evidence for our understanding of the target modulation mechanism of EA analgesia.

#### 4.2.2. Dopamine

DA is one of the major neurotransmitters in the CNS, transmitting signals through five G-protein–coupled receptors from D1 to D5. Dopamine receptors are widely expressed in the brain, especially in the midbrain dopamine reward system, contributing to downstream pain and regulation of negative emotions. For example, dopamine receptors D1 and D2 in the ACC play different roles in the regulation of trigeminal pain, and optogenetic activation of D1-expressing neurons significantly exacerbated CCI-ION-induced trigeminal pain, but optogenetic activation of D2-expressing neurons ameliorated this pain (Liu et al., 2020). In contrast, one study found that protein levels of DRD1 were significantly reduced, and DRD2 protein levels were elevated in the BLA of SNI mice. EA stimulation of the ZuSanLi (ST36) and SanYinJiao (SP6) acupoints significantly increased the levels of DRD1 protein in the amygdala, decreased the levels of DRD2 protein, and attenuated SNI-induced anxiety-like behaviors but had no effect on mechanical anomalies of pain. These results suggest that EA attenuates anxiety-like behaviors in SNI mice by activating DRD1 or antagonizing DRD2 in the BLA but is not involved in alleviating SNI-induced NP (Wu et al., 2022).

In addition, DA is an important neurotransmitter and a crucial anti-inflammatory mediator in peripheral and central inflammation. DRD1 and DRD2 are the primary pharmacological targets of neuroinflammation (Yan et al., 2015; Zhu et al., 2018; Cheng et al., 2020). EA treatment attenuated CCI-induced neuroinflammation in the amygdala while upregulating DA concentration and DRD1 and DRD2 expression in the amygdala, ultimately relieving pain and negative emotions (Zhang X. H. et al., 2021).

Taken together, this suggests that the effects of EA on pain sensation and mood in NP may be related to the regulation of the DA system, but the roles of dopamine receptors D1 and D2 in NP in different brain regions are controversial and need to be further verified by larger scale experiments.

#### 4.2.3. Acetylcholine

The central cholinergic system has an essential role in attention, spatial learning, and memory, as well as in nociceptive modulation and analgesia. Acetylcholine (ACh) is a vital neurotransmitter or modulator in this pathway and acts on the CNS mainly by mediating the nicotinic acetylcholine receptor (nAChR) and muscarinic acetylcholine receptor (mAChR). Injection of ACh at the ZuSanLi (ST36) acupoint produces acupuncture-induced analgesia for inflammatory or NP (Vieira et al., 2018), suggesting that acetylcholine receptors can be a target of action for EA against NP.

In addition, the results of many animal experiments have shown that modulation of the cholinergic system is one of the mechanisms by which EA treats NP. In rat models of CCI-induced NP and ovariectomy-induced learning and memory impairment, the expression of acetylcholinesterase, vesicular acetylcholine transporter, and muscarinic M1 receptor mRNA in the hippocampus and hypothalamus was increased, and the pain threshold was decreased after repeated EA stimulation of ST36-GB34. The results suggest evidence that both hippocampal and hypothalamic cholinergic activity are involved in cumulative analgesia induced by repeated EA in CCI rats (Wang J. et al., 2012; Wang J. Y. et al., 2012). Injection of mAChR and nAChR antagonists into the hippocampus, respectively, abolished the analgesic effects of EA stimulation, further confirming the involvement of mAChR and nAChR in the analgesic results of EA (Chen et al., 2016).

#### 4.2.4. Opioid peptides

At the central level, the analgesic effects of EA stimulation are also closely related to opioid peptides and their receptors. The main endogenous opioid peptides that have been studied include enkephalin, endorphin, and orphanin-FQ. For example, EA can exert analgesic effects by enhancing orphanin-FQ immunoreactivity and decreasing precursor protein FQ mRNA expression in the NRM of rats with CCI NP (Ma et al., 2004). Similarly, in a CCI model, EA of bilateral foot-sanli and Yanglingquan significantly increased the content of amygdala μ-opioid receptors, suggesting that EA can activate opioid peptides in the nucleus accumbens, which are involved in the downward inhibitory system (Yan et al., 2016). One study evaluated the analgesic effect of EA with different combinations of parameters in SNL rats, in which 2/120 Hz EA applied at the ZuSanLi and KunLun points significantly reduced the NP of rats and increased the content of enkephalin and β-endorphin in the hypothalamus, suggesting that the analgesic effect of EA in SNL rats is related to the expression of opioid peptides in the hypothalamus (Zhou et al., 2017).

#### 4.2.5. 5-HT

5-HT has also been reported to be involved in treating NP by acupuncture. In the CNS, 5-HT has a bi-directional regulatory role and EA can exert analgesic effects by up-and down-regulating the expression of 5-HT and its receptors. In CCI mice, EA treatment increased mechanical nociceptive hypersensitivity and thermal abnormal pain thresholds, improved CCI-induced depressive-like behavior, and significantly reversed the down-regulation of the expression levels of 5-HT in the ACC and spinal cord after CCI, suggesting that the analgesic and antidepressant effects of EA may be mediated by up-regulation of 5-HT in the ACC and spinal cord of mice (Cong et al., 2021). Carcinomatous bone pain is a chronic pain involving both inflammatory and NP. Wrist-ankle acupuncture and EA attenuates mechanical pain hypersensitivity in rats with carcinomatous bone pain by inhibiting the expression of 5-HT and 5-HT3aR in the downstream RVM-spinal circuit, suggesting that 5-HT plays a vital role in the downstream control of pain in the CNS (Zhang et al., 2020).

#### 4.2.6. Orexiu

The orexins, also known as hypocretin, are divided into two subtypes, namely orexin A and orexin B, which modulate pain perception at the spinal cord and supraspinal levels (Razavi and Hosseinzadeh, 2017). Some studies have suggested that orexins may mediate the analgesic effects of EA at the supraspinal level. Orexin neurons are predominantly located in the hypothalamus, especially the LH. Using the conditioned place preference paradigm, Wang et al. (2022) found that EA produced a rewarding effect in early pain relief and activated c-Fos-positive orexin neurons in the LH and LH-NAc circuits. Microinjection of an orexin-A antagonist in the LH-NAc blocked the reward induction of EA. This suggests that appetitive hormone signaling in the LH likely plays a key role in the rewarding effects of EA-induced pain relief (Wang et al., 2022). The PAG is also an important supraspinal site for EA-mediated appetitive hormone pain modulation. Chen et al. (2018) revealed a non-opioid analgesic mechanism induced by electrical stimulation of the median nerve at the NeiGuan (MNS-PC6 Similarly, Lee et al. (2021) further found in opioid-tolerant mice with CCI-induced NP that EA at SP6 could induce activation of hypothalamic orexin neurons through activation of cannabinoid receptor 1, which mediates de-inhibition of PAG and ultimately effectively promotes EA analgesia. Thus, EA can modulate supraspinal orexin and its receptor interactions independently of the opioid system to significantly reduce pain.

In addition, extracellular ATP facilitates pain transmission at peripheral and spinal sites via P2X receptors. It was found that in NP states, the pain threshold of lPAG was decreased and P2X3 receptor expression was upregulated. Multiple EA treatments increased the pain threshold and increased the immunoreactivity of P2X3 receptors in lPAG in rats with NP, and down-regulation of P2X3 receptor expression in lPAG significantly attenuated the anti-injury sensory effects of EA treatments. These results suggest that P2X3 receptors in lPAG inhibit pain modulation and that the therapeutic effect of EA on NP in CCI rats may be related to its modulatory effect on lPAG P2X3 receptor expression (Xiao et al., 2010). In conclusion, P2X3 receptors in lPAG are also involved in EA therapy’s supraspinal anti-injury sensory effects.

Taken together, the above studies show that EA modulates neurotransmitters such as glutamate, GABA, dopamine, acetylcholine, and related receptors in higher brain regions to alleviate NP and induce negative emotions and cognitive deficits. Thus, it is significant to carry out the studies by targeting neurotransmitters and receptors to explore the mechanism of EA-based analgesia.

### 4.3. Acupuncture treats NP by affecting brain metabolism

More than 95% of ATP in the brain is provided by glucose metabolism; hence, glucose metabolism can reflect the functional state of the brain to some extent. Recently, several papers have focused on the functional abnormalities of the brain in NP states and found that glucose metabolism in several brain regions such as the hippocampus (Thompson et al., 2014), somatosensory cortex (Shen et al., 2019a), thalamus (Shen et al., 2019b), mPFC, and ACC (Lin et al., 2014) underwent significant changes. This suggests that the homeostasis of glucose metabolism in the brain is closely related to the development of NP.

Acupuncture can exert analgesic effects by modulating glucose metabolism in brain regions. It was found that the expression of GLUT-3 in the hippocampus and cortical neurons of CCI rats could be up-regulated by EA to the acupoints of HuanTiao (GB30) and YangLingQuan (GB34), which increased glucose metabolism in the brain region, thereby alleviating the hyperalgesia reaction (Jiang M. et al., 2021). Furthermore, in the CCI rat model, it was observed that after EA stimulation of ZuSanLi (ST36) and YangLingQuan (GB34), there were significant changes in the expression of 17 proteins in the hypothalamus, in particular, considerable modulation of glycolysis/gluconeogenesis/hexose metabolism pathway. These data suggest that EA interventions can reduce pain by modulating the expression of multiple proteins in the hypothalamus and that hypothalamic glucose metabolism may play an important role in energy support (Gao et al., 2013). 18FDG-PET was used to assess changes in brain metabolic connectivity in an NP model before and after EA stimulation of cervical “Jiaji” acupoints (EX-C5-C7). The metabolic brain connectivity between the somatosensory cortex, motor cortex, caudate putamen, and dorsolateral thalamus in bilateral hemisphere was reduced in the BPAI group. The strength of connectivity between the above regions was significantly increased after 4 and 12 weeks of EA, suggesting that modulation of cerebral metabolic connectivity may be an important mechanism for the analgesic effect of EA in NP (Hou et al., 2020).

In conclusion, the changes in brain glucose metabolic activity during the analgesic action of acupuncture in neuropathic rats suggests that brain regions such as the hippocampus, mPFC, hypothalamus, thalamus, and somatosensory cortex may be the main sites of EA analgesic action and that by identifying the changes in glucose metabolism of these action targets of NP, we can better interpret the acupuncture analgesia mechanism and design more accurately targeted therapies for NP.

### 4.4. Acupuncture treats NP by inhibiting nerve inflammation

Excessive neuroinflammation is a fundamental mechanism that generates and maintains chronic NP (Lees et al., 2015). During nerve injury or neurological inflammation, the secretion and release of neuroinflammatory mediators induce and modulate NP by promoting peripheral and central sensitization, including interleukins, tumor necrosis factor (TNF), and HMGB1 (Wan et al., 2016; Sommer et al., 2018; Fumagalli et al., 2020). Therefore, actively targeting pro-inflammatory mediators in the spinal cord at critical time points may be a novel approach to treating NP.

For example, one study explored the role of EA in relieving abnormal mechanical pain by differential proteomic analysis of the hippocampus of rats with NP, which not only identified differentially expressed proteins involved in EA analgesia but also verified that the hippocampal protein, TMEM126A, exerts an important anti-inflammatory role in EA for NP (Zhang X. H. et al., 2021). In the CCI rat model, the hippocampus’s expression of tumor necrosis factor-alpha (TNF-α), and interleukin-1beta (IL-1 β) was elevated. In contrast, EA stimulation could substantially down-regulate the levels of these pro-inflammatory factors in the hippocampus while alleviating the pain in rats. It is suggested that EA can exert analgesic effects by decreasing specific pro-inflammatory mediators in the hippocampus (Wang et al., 2018).

Excess pro-inflammatory mediators in certain supraspinal structures can also mediate the emergence of affective disorders in NP states (Fiore and Austin, 2016). In a pain-depression co-morbidity model of chronic unpredictable mild stress with CCI, there was a significant increase in the expression of TNF-α in the plasma and related brain regions, including the PFC, hippocampus, amygdala, and hypothalamus, and the EA interventions suppressed the elevated levels of TNF-α. These results suggest that the antidepressant and analgesic effects may be related to the inhibition of TNF-α–related neuroinflammation (Guo et al., 2020).

Overactive astrocytes and microglia are also major causes of neuroinflammation. Microglia and astrocytes are widespread and involved in the spinal cord and brain. After nerve damage, they are rapidly activated and produce many inflammatory mediators involved in the neuroimmune inflammatory process that causes pain hypersensitivity. Zhang et al. showed that CCI induced significant activation of astrocytes and elevated levels of TNFα and IL-1β protein in the amygdala of rats, whereas microglial cells remained unaffected. EA treatment significantly improved CCI-induced abnormal pain and emotional dysfunction, reducing the amygdala’s TNFα, IL-1β, and glial fibrillary acidic protein (Zhang X. H. et al., 2021). In the SNI-induced NP model, EA could improve NP and depressive behavior, inhibit the proliferation and activation of microglia in the dorsal horn of the spinal cord and hippocampus, and reduce neuroinflammation. These results suggest that the improvement of NP and negative mood by EA may be related to the inhibition of the over-activation of glial cells in certain brain regions, which in turn reduces neuroinflammation in brain regions.

### 4.5. Acupuncture mediates signal transduction pathways for NP treatment

In recent years, more and more studies have focused on signaling pathways to explore the mechanism of NP. Pain information initiates central sensitization through different signaling pathways, and targeting signal-transducing molecules distributed on the pathways to inhibit the transmission of pain information will be beneficial to reduce the onset of NP. Studies have confirmed that acupuncture can mediate different signal transduction pathways or signal transduction molecules to exert analgesic effects, mainly including the mitogen-activated protein kinase (MAPK) signaling pathway, nitric oxide (NO) signaling pathway, and other pathways.

#### 4.5.1. MAPK signaling pathway

One study analyzed the overall changes in relevant proteins in the hippocampus after acupuncture (ST36 and GB34) interventions in NP conditions, and differential protein enrichment analyzes suggested that the analgesic effect of EA was mediated through the modulation of hippocampal amino acid metabolism-related proteins and activation of the MAPK signaling pathway (Gao et al., 2012).

MAPK is an important signaling pathway that mediates the transmission of external stimuli to the intracellular space, consisting of extracellular-regulated protein kinase (ERK) 1/2, c-Jun N-terminal kinase 1/2/3, p38 isoforms (α, β, γ, and δ), and ERK5, which collectively mediate a range of activities including proliferation, differentiation, apoptosis, immunity, and inflammation. In NP states, MAPKs are widely activated in the spinal cord, dorsal root ganglia, and brain and play an important role in NP’s central and peripheral sensitization (Crown, 2012; Hu et al., 2017; Zhang et al., 2019; Yan et al., 2022), suggesting that MAPKs could be an essential target for treating NP (Ma and Quirion, 2005). Wang et al. (2015) further found that EA stimulation of ST36 and GB34 reversed the expression of ERK1 and p-ERK1/2, p38 MAPK, and phosphorylation in hippocampal tissues, suggesting that EA can be used to alleviate abnormal post-CCI pain by modulating hippocampal ERK and p38MAPK signaling pathways. In CCI rats, acute injurious stimulation takes longer to restore the firing rate of pain-related neurons to normal levels; EA treatment inhibits the effects of harmful stimuli on PENs and PINs in CCI rats (Wang et al., 2016), and intrahippocampal injection of ERK-specific inhibitors antagonizes the effects of EA on analgesic pathways. It is suggested that the activation of the ERK signaling pathway in the hippocampus involved in EA treatment can induce pain relief (Gao et al., 2016; Wang et al., 2016). The intense stimulation of the “HuanTiao” (GB 30) by hand needling effectively alleviated the pain-anxiety effect in a rat model of neuropathic pain, which was associated with the down-regulation of p-ERK expression in the ACC (Shao et al., 2015), suggesting that the ERK signaling pathway is involved in not only the sensory dimensions of pain and EA analgesia but also in the emotional dimensions of pain.

#### 4.5.2. No signaling pathway

Nitric oxide (NO) is a gaseous messenger molecule with neurotransmitter-like functions and has been shown to be involved in generating and processing injury sensations (Salter et al., 1996; Luo and Cizkova, 2000). Endogenously synthesized and exogenously administered NO is involved in CCI-induced neuropathy in rats. The significant increase in nitrate and nitrite levels in ligated sciatic nerves also suggests local upregulation of NO in NP (Onal et al., 2003; Naik et al., 2006). NO plays an important role in producing and maintaining NP (Yoon et al., 1998).

Endogenous nitric oxide is formed by three isoforms of nitric oxide synthase (NOS): inducible NOS (iNOS), neuronal NOS (nNOS), and endothelial NOS (eNOS). Studies on the localization of NOS have shown that it is present in almost all brain regions (Blum-Degen et al., 1999). Knockdown and pharmacological inhibition of nNOS reduce pain sensitivity in animal models of NP (Guan et al., 2007; Staunton et al., 2018). The above suggests that the NO/NOS signaling pathway is involved in the pathological process of NP (Meller et al., 1992).

EA can exert analgesic effects by mediating this signaling pathway. The pain threshold of rats with postherpetic neuralgia increased continuously after EA treatment, accompanied by decreased NO content and iNOS activity in the brain tissue. Thus, one of the mechanisms of EA in alleviating neuropathic pain may be related to its effective regulation of the NO/iNOS transduction pathway in the brain tissue of rats (Jiang K. Y. et al., 2021). Another report found that in the CCI-induced NP rat model, the expression of nNOS and *PKG* genes in the hippocampus was significantly elevated. However, the expression of iNOS did not change significantly, and the expression of nNOS and *PKG* genes was reduced considerably after EA stimulation at different frequencies, suggesting that the hippocampal nNOS/PKG signaling pathway may be involved in the cumulative analgesic effect produced by acupuncture in a chronic pain rat model (Kan et al., 2013).

#### 4.5.3. EphrinBs/EphBs signaling pathway

EphBs is a member of the tyrosine kinase subfamily that binds to its ligand EphrinBs to form the EphrinBs/EphBs signaling pathway, which is involved in the modulation of detrimental spinal cord messages and central sensitization, and the activation of the spinal cord EphrinBs/EphBs system has an important role in the formation and maintenance of NP (Ruan et al., 2010; Yu et al., 2012). For example, in the dorsal root ganglia and spinal cord, expression of ephrinB1 and EphB1 receptor proteins was significantly upregulated in a time-dependent manner and corresponded to the development of thermal nociceptive hypersensitivity after CCI (Song et al., 2008).

Similarly, Eph receptors with their ligand Ephrin are widely expressed in the hippocampus. EphB1, EphB2, EphB3, and EphrinB2 mRNA expression levels were significantly increased in the hippocampal tissues of CCI model rats, suggesting that alterations of these genes in the hippocampal region may be involved in the development of NP. Meanwhile, long-term acupuncture significantly suppressed the mechanical nociceptive hypersensitivity behavior of rats with NP and widely regulated the expression of genes related to the EphrinBs/EphBs system in the hippocampus, which may be one of the mechanisms for the treatment of NP by acupuncture (Wang et al., 2014). However, the mechanism by which acupuncture analgesia modulates the cellular signal transduction pathway of the relevant EphrinBs/EphBs system has been less studied, and further studies are still needed.

In summary, MAPK, NO, and EphrinBs/EphBs-related signaling pathways are closely related to the pathogenesis of NP, and the mechanism of action of EA in NP treatment can be further investigated based on these signaling pathways. Of course, many studies have shown that EA can be involved in the regulation of NP through DRG and other signaling pathways in the spinal cord, such as Nrf2 antioxidant signaling pathway (Zhao et al., 2019), A2AR/cAMP/PKA signaling pathway (Wu et al., 2021), and cAMP-PKA-CREB signaling pathway (Yan et al., 2018), but there is a lack of intracerebral related studies at present.

### 4.6. Acupuncture for NP via other routes

The underlying mechanism by which acupuncture treatment works in relieving NP is complexand by no means limited to modulating synaptic plasticity, regulating glucose metabolism in brain regions, and reducing neuroinflammation. For instance, it was found that EA stimulation of the ZuSanLi (ST36) and SanYinJiao (SP9) down-regulated the expression of miR-34a and p53 in the contralateral cerebral cortex of CCI mice, which mediated the anti-nociceptive hypersensitivity effect (Chen et al., 2019). In addition to the analgesic effects, acupuncture may improve neuropathic pain-induced mood/cognitive dysfunction by restoring overall DNA methylation in the PFC (Jang et al., 2021). Li S. et al. (2023) concluded that EA at BaiHui and YinTang could alleviate pain and depressive behavioral symptoms in SNI-induced pain-depression model rats, and its analgesic and antidepressant mechanism might be related to the up-regulation of hippocampal AcH3 and BDNF expression. One study investigated the intervention mechanism of different acupuncture methods on negative emotions in rats with neuropathic mirror pain, which may be related to the inhibition of proliferative activation of ACC microglia (Shen et al., 2016). In addition, hyperpolarization-activated cyclic nucleotide-gated channels 1 (HCN1) in the affected habenular nucleus of rats were also involved in the development of NP associated with anxiety and interfered with the expression of HCN1 in the habenular nucleus, which may be a possible means by which EA regulates NP accompanied by mental disorders (Peng et al., 2020). In conclusion, these findings suggest that acupuncture can also affect miRNA expression in different brain regions, DNA methylation, microglia proliferation, ion channels, and other multiple pathways to exert their analgesic effects.

## 5. Discussion

Neuropathic pain is a complex physiological-psychological sensation involving pain sensation, pain emotion, and pain cognition. With the help of different animal models of NP, we have summarized the brain regions and neural circuits related to pain and the brain mechanisms involved in pain regulation by EA. We found that NP involves the participation of different brain regions and nuclei, such as PAG and RVM, which are responsible for the pain sensation, and ACC and PFC, which are responsible for the pain emotion/cognition dimension. However, the brain regions responsible for the various pain dimensions are not isolated, and they project networks among each other to mediate the development of NP. In addition, needling information and other harmful signals can reach different brain regions and affect their functional activities via multiple targets, angles, and links to provide analgesia, including restoring synaptic plasticity, regulating brain metabolism, decreasing neuroinflammatory responses, affecting the secretion and release of neurotransmitters and receptors, and mediating different signaling pathways. To a certain extent, these studies have significantly deepened our understanding of the mechanism of NP and the analgesic mechanism of acupuncture, but some limitations still exist.

Current research on pain-related neural circuitry or acupuncture analgesic modulation mechanisms relies mainly on animal models. However, the criteria for constructing animal models are not standardized, and conclusions based on different models often differ or are even contrary. In addition, unlike humans, experimental animals cannot respond to the degree of pain or emotion with the help of words or expressions. Their pain can only be assessed with the help of some single behavioral methods. Therefore, pain models should be further improved, corresponding rubrics should be developed according to different model types, different data should be reported and interpreted in the context of specific NP models, and these kinetic preclinical data should be translated into clinical pain management applications as much as possible. In addition, future clinical studies should emphasize on exploring issues such as the effects of acupuncture on functional changes in brain regions and brain circuits in patients with NP. For example, changes in the functional connectivity of the brain in patients with NP and acupuncture treatment should be explored with the help of neuroimaging and other techniques to identify the brain neural circuitry associated with acupuncture analgesia.

The brain nuclei behind NP and the interwoven neural circuits are complex. As research has gradually progressed, even the same brain nucleus shows different responses to painful stimuli. For example, the conventional view is that pain inhibits VTA DAergic neuronal activity and reduces DA release. However, in recent years, increased DAergic neuronal activity in the VTA can exacerbate the pain state. The reasons for this may be related to the interaction of multiple neurons in the VTA, the existence of local microcircuits between different subregions, and the existence of parallel projections between the VTA and other nuclei, and further studies are needed to address these aspects. In addition, PAG-RVM is thought to mediate pain descending inhibition and facilitation and regulate NP in both directions. Hence, more research evidence is needed to clarify interior reasons.

At the neural level, research on the analgesic mechanism of acupuncture for NP has yet to catch up with research on its pathogenesis. For example, research on the analgesic mechanism of acupuncture is still limited to a single nucleus, such as the hippocampus, amygdala, and anterior cingulate gyrus, and there needs to be more research on the modulation of the neural circuits in the brain. Moreover, research on the analgesic mechanism of acupuncture is more inclined to longitudinal refinements, such as acupuncture as it can regulate a variety of neurotransmitters and receptors in the brain tissue to play an analgesic effect. Of these, some neurotransmitters play a role in the hippocampus, and some play a role in the amygdala, but the coordination of each other’s role in the study of the lesser. Therefore, the focus of future research can be appropriately directed toward cerebral neural circuits; at the same time, it is also necessary to pay attention to the lateral connection and explore what kind of coordination mode is involved in EA-based analgesia between different neurotransmitters in the same brain area, or the same neurotransmitter in different brain areas. In addition, acupuncture for NP is complicated, and there is a big difference in the selection of acupoints and frequency of acupuncture in different animal models and even in the same model. Therefore, in the future, it is necessary to develop a unified principle of selecting acupuncture points for various causes of NP and, at the same time, to verify the results of clinical trials with modern science and technology to provide a systematic and scientific research method for the selection of acupuncture points for treatment of NP in the clinic.

## Author contributions

NS and PC searched literature and wrote the article. ZD, XY, HX, and JH collected data and drew figures. ZL and CL designed article ideas and performed language embellishment. All authors contributed to the article and approved the submitted version.

## Funding

This article was supported by Key Research and Development project of Shandong Province (No. 2019GSF108197); Rongxiang Regenerative Medicine Foundation of Shandong University (No. 2019SDRX-21); Jinan Science and Technology Program (202019161); Acupoint-Viscera Correlation Study Youth Scientific Research Innovation Team, Shandong University of Traditional Chinese Medicine (22202110).

## Conflict of interest

The authors declare that the research was conducted in the absence of any commercial or financial relationships that could be construed as a potential conflict of interest.

## Publisher’s note

All claims expressed in this article are solely those of the authors and do not necessarily represent those of their affiliated organizations, or those of the publisher, the editors and the reviewers. Any product that may be evaluated in this article, or claim that may be made by its manufacturer, is not guaranteed or endorsed by the publisher.
